# Silicone migration to the contralateral axillary lymph nodes and breast after highly cohesive silicone gel implant failure: a case report

**DOI:** 10.1186/1757-1626-2-6420

**Published:** 2009-03-10

**Authors:** Gabriel J Kaufman, Rita A Sakr, Cyrille Inguenault, Isabelle Sarfati, Claude Nos, Krishna B Clough

**Affiliations:** 1 Department of breast cancer and reconstructive surgery, Institut du Sein, Paris Breast Center, 7 Avenue Bugeaud, 75116 Paris, France

## Abstract

Highly cohesive silicone gel implants are advertised for aesthetic and safety advantages. Our case is the fourth report describing early implant rupture and contralateral migration of siliconoma. Despite the greater degree of gel cohesiveness, a continued vigilance for signs and symptoms of migration is highly recommended.

## Introduction

The introduction of highly cohesive silicone gel implants (HCGI) advertised favorable aesthetic and safety advantages over standard cohesive gel implants. These included greater durability of overall shape particularly with regards to the upper-pole volume and a reduction in incidence of outer shell folding. The safety profile also improved with the greater degree of gel viscosity by limiting migration and loco-regional spread of silicone gel after compromise of the implant shell. Since the introduction of HCGI in 1993 there have only been 3 published case reports of regional spread and axillary lymph node involvement after capsular rupture of an HCGI [1-3].

## Case presentation

**Figure 1:**
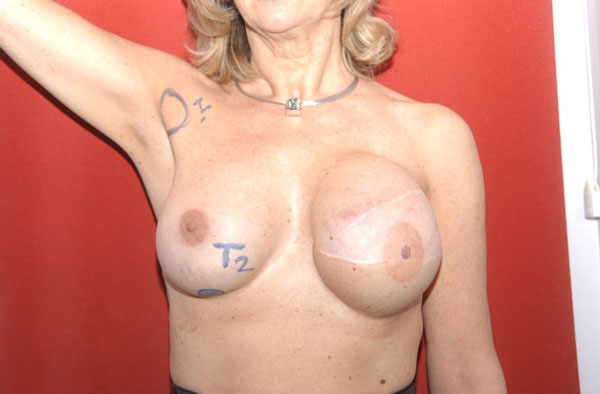
**Early enlargement of the patient's left reconstructed breast**.

**Figure 2:**
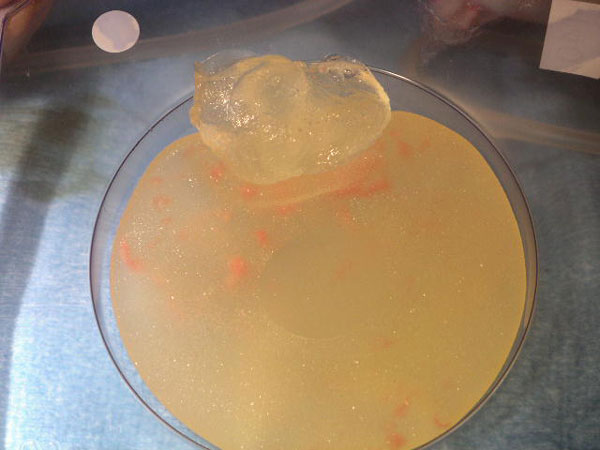
**Implant rupture with extrusion of the high cohesive gel into the upper pole**.

An European Caucasian 59-year-old patient had delayed reconstruction with a latissimus dorsi flap and McGhan 410 highly cohesive silicone implant after a modified radical mastectomy of the left breast. Prior to reconstruction, the patient was treated for multifocal invasive ductal carcinoma with adjuvant chemotherapy and radiation to the chest. During reconstruction, symmetrization of the right side was achieved by performing a superior pedicle mammoplasty and insertion of a Poly Implant Prosthesis (PIP) gel implant. After 2 years of routine follow-up, the patient experienced rapid enlargement of her reconstructed left breast (Figure 1). Findings were suspicious for implant rupture and seroma formation, however; a palpable mass of the augmented right breast was also noted on examination as well as right axillary lymphadenopathy. Biopsy was performed on both the right breast mass palpable axillary node to rule out malignancy. The biopsy demonstrated findings consistent with siliconoma. Axillary dissection revealed 3 large rubbery nodes, the greatest measuring approximately 2 cm in diameter. A capsular mass was identified on the right side and the implant shell appeared to be intact. Examination of the left breast demonstrated seroma and implant rupture with extrusion of the high cohesive gel into the upper pole (Figure 2). The left and right implants were removed and both replaced with PIP standard profile silicone implants. Final pathology was consistent with siliconoma for both the enlarged lymph nodes and right breast mass.

## Discussion

Silicone gel entering the lymphatics, either through overt implant rupture or slow leakage across the intact outer shell, can result in regional migration to the draining lymph node basins [1,2]. Axillary lymphadenopathy in any patient with a history of breast cancer should raise concern for recurrence and prompt aggressive evaluation to avoid delays in diagnosis. Migration of silicone is not always limited to the corresponding axillary lymph nodes and spread to the internal mammary and inguinal nodes as well as the abdominal wall and lower back have all been reported in the literature [1-4]. Our report is the first to describe a silicone granuloma within the capsule of the contralateral breast and axillary lymph nodes.

With the introduction of highly cohesive silicone gel matrix implants in the 1990's the risk of local-regional spread after rupture was thought to have been ameliorated. The early experience with highly cohesive implants resulted in low complication rates without evidence for silicone migration [1].

The 3 year results of the highly cohesive silicone breast implant core study reported a less than 1% device rupture rate [2]. Magnetic resonance imaging (MRI) was used to evaluate patients for evidence of rupture in this study. An additional European series from Sweden found a 0.3% incidence of rupture based on MRI evaluation, thus confirming low rupture rates in this type of implant [3]. The causes of implant rupture are varied, but those commonly reported are compression from closed capsulotomy, mammography or trauma with the actual cause often unknown [4].

MRI has proven to be sensitive in the detection of implant rupture. Comparison studies have demonstrated higher rates of sensitivity using MRI compared to mammography or ultrasonography when the appropriate breast coil is utilized [5]. The role of fine needle aspiration for palpable lesions in the axilla and breast after breast augmentation is a useful tool in differentiating between cancer recurrence and silicone granulomas [6].

## Conclusion

Early implant failure of HCGI is rare, but despite the increased gel viscosity the potential for regional migration remains. This is the fourth case report describing regional migration. Our case report adds to a growing awareness of this phenomenon and emphasizes the need for continued vigilance for signs and symptoms of migration despite the greater degree of gel cohesiveness.

## Abbreviations

HCGI: Highly cohesive silicone gel implants; PIP: Poly Implant Prosthesis; MRI: Magnetic resonance imaging.

## Consent

Written informed consent was obtained from the patient for publication of this case report and accompanying images. A copy of the written consent is available for review by the Editor-in-Chief of this journal.

## Competing interests

The authors declare that they have no competing interests.

## Authors' contribution

GK and RS performed the writing of the manuscript. CI, IS and CN contributed to analysis. KC contributed to revision, supervision and approval of the work.
